# Force accuracy rather than high stiffness is associated with faster learning and reduced falls in human balance

**DOI:** 10.1038/s41598-020-61896-1

**Published:** 2020-03-18

**Authors:** Amel Cherif, Ian Loram, Jacopo Zenzeri

**Affiliations:** 1 Department of Robotics, Brain and Cognitive Sciences, Istituto Italiano di Tecnologia, Via Enrico Melen, 83, 16152 Genoa, Italy; 2 Department of Informatics, Bioengineering, Robotics, and System Engineering, University of Genoa, Via All’Opera Pia, 13, 16145 Genoa, Italy; 3 Cognitive Motor Function Research Group, Research Centre for Musculoskeletal Science & Sports Medicine, Dept of Life Sciences, Faculty of Science and Engineering, Manchester Metropolitan University, Manchester, M1 5GD UK

**Keywords:** Motor control, Sensorimotor processing

## Abstract

Balance requires the centre of mass to be maintained within the base of support. This can be achieved by minimising position sway (stiffness control: SC) or minimising force error (force accuracy control: FAC). Minimising sway reduces exploration of system properties, whereas minimising force error maximizes accurate mapping of the force vs position. We hypothesise that (i) FAC is associated with faster learning and fewer falls whereas (ii) SC is not. Fifteen participants used myoelectric signals from their legs to maintain balance of an actuated, inverted pendulum, to which they were strapped. Using challenging perturbations, participants were trained to maintain balance without falling within five sessions and tested before (PRE) and after (POST) training. We quantified FAC as ‘change (POST-PRE) in correlation of force with position’ and SC as ‘change in sway’. PRE training, five measures (sway, acceleration, co-contraction, effort, falls) showed no correlation with either FAC or SC. POST training, reduced fall rate, effort and acceleration correlated with FAC metric. SC correlated only with reduced sway. Unlike sway minimisation, development of force accuracy was associated with learning and reduced falls. These results support that accurate force estimation allowing movement is more relevant than stiffness to improve balance and prevent falls.

## Introduction

Postural balance requires the whole body centre of mass (CoM) to be maintained within the base of support during both self-initiated and externally triggered disturbances of stability. In human postural sway, the dynamic relationship between the combined sagittal ankle joint moment and sagittal position of the CoM is similar to the control of an unstable, inverted pendulum^1,2^. Since the passive stiffness of the ankle joint is lower than the growth-rate of the gravitational toppling torque, an active feedback control mechanism is needed for upright standing^3,4^. Human control of an external, unstable inverted pendulum using the postural lower leg muscles (calf, and tibialis anterior) provides a balance task which replicates a key component of postural standing balance^5^.

**Figure 1:**
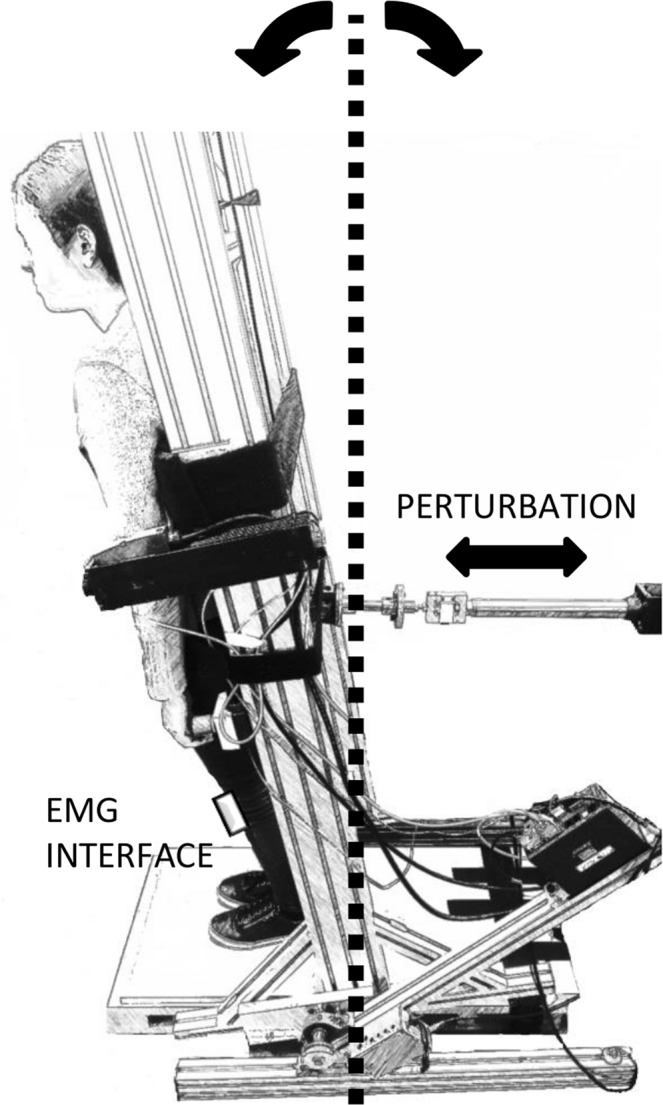
Experimental setup. The participant stands upright with feet on a stationary footplate, rigidly strapped to the apparatus. The WBM is actuated myoelectrically: sEMG signals from both legs TA and G are acquired and used to generate the control signal. Hence passive joint properties play no role in this task. WBM provides to the participant haptic feedback of the controlled inverted pendulum position, rotating around its virtual ankles. The angular position is null when the WBM is vertical. The neutral position was set 2° forward, according to physiological standing. This 2° position requires a small, tonic plantar-flexion moment to maintain balance which adds to the natural feel of the task.

In order to study the process of postural balance, participants were strapped to, and controlled an actuated system, with an unstable (inverted pendulum) time constant of a typical human body. In this experiment, the sensory feedback, the motor action and the ownership of self-movement ensure the task feels very similar to postural balance (Supplementary Videos 1–2). The system was controlled by a torque determined by activations of the calf and tibialis muscles of the participants (Fig. 1). This linearized inverted pendulum system is represented by an equation of motion including two parts (1).

1 $$T=-\,mgh{\theta }+{\rm{I}}\ddot{{\theta }}$$

The first part is a linear relationship between ankle moment *T* and pendulum angle θ, where *m* is the mass of the pendulum, *g* is the gravitational acceleration, *h* is the distance between the centre of mass and the joint and *I* the moment of inertia around the centre of mass. Deviation from the linear relationship (second part of the equation) represents the acceleration and also represents the torque error from the equilibrium torque required for the angle of the pendulum. When plotted as torque versus angle, the best-fit line indicates the torque vs angle relationship of the inverted pendulum and torque differences/errors from this ideal line are related to acceleration^6^. Postural balance requires the person to prevent falls which in turn requires the participant to maintain an appropriate torque for any angle and also to regulate the angle within limits of stability (corresponding to the base of support). To prevent falls there are many possible feedback mechanisms and strategies.

Previous studies showed that humans adopt a strategy from a spectrum of choices ranging from two extremes which we describe as minimising variance in position (stiffness control: SC) or minimising force error (force accuracy control: FAC)^6–9^. This spectrum of choice is fundamental within control theory. Within the optimal feedback control framework this choice is represented within the cost function as prioritising regulation of position or regulation of effort^10,11^.

For unstable tasks involving the upper limbs, two extreme strategies were observed^7,8,12^. The first strategy (high-stiffness strategy) implies the production of a convergent, restoring force field, taking advantage of the elastic properties of the body/environment system. This can be a successful strategy but it has two main disadvantages: it works only if body stiffness is greater than the rate of growth of the divergent field and it is energetically expensive. The second strategy (low-stiffness strategy), instead, is based on explicit positional feedback from different sensory channels (e.g. proprioception and vision). These strategies have also been identified by Loram *et al*.^5^ who studied human control of an inverted pendulum.

Therefore, from previous literature we focus attention on two strategy extremes:

- Stiffness control strategy (SC), which consists in minimising position sway.
- Force accuracy control strategy (FAC), achieved minimising force error: a high weight given to sensory feedback, with minimal acceleration.

**Figure 2:**
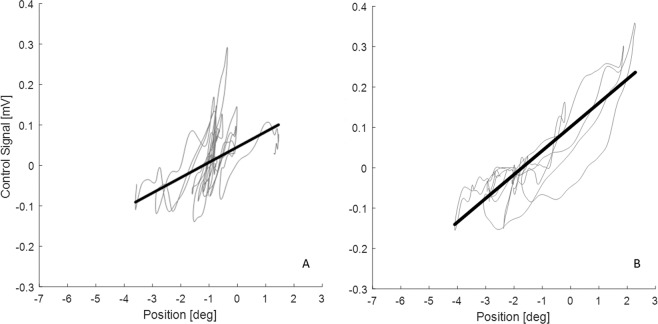
Representative examples of the two control strategies. (**A**) Stiffness control strategy (SC). (**B**) Force accuracy control strategy (FAC). In example (**A**), the fluctuation on control signal (ankle torque) is quite large and does not follow the equilibrium control signal v position line very closely. In example (**B**) the control signal does follow the equilibrium value closely for range of positions and hence the correlation between control signal and position is higher than in example (**A**).

These extremes are illustrated in Figure 2A, represents a variation of forces unrelated with the relatively narrow range of positions. Figure 2B shows a more accurate mapping of the force vs position and a wider range of positions.

In postural control, the consequences of the choice within the spectrum of possibilities (SC to FAC) for learning and for fall prevention remain unknown. For postural control high variance in position (sway) has been viewed as a sign of poor balance control and increased fall risk^13,14^. Alternatively, according to the exploratory hypothesis^15–17^, postural sway is viewed not as pure noise in the postural control system, but as part of a perception-action strategy that allows humans to gain essential information about their interaction with the environment.

Minimisation of position variance (SC) per se, does not require ability to maintain appropriate torque for any angle. The ability to maintain an appropriate torque for any angle (FAC) requires greater control facility than the ability to minimise position variance, since FAC requires regulation of force in addition to regulation of position. Arguably, FAC requires implicit knowledge of the torque vs angle relationship of the inverted pendulum. Attaining FAC requires a learning process. Following the previous literature, we hypothesize (i) that minimising sway (SC) reduces exploration of system properties such as the force vs position relationship and is associated with poor learning and limited reduction in falls; and (ii) that minimising force error (FAC) maximizes accurate mapping of the force vs position and it is associated with faster learning and fewer falls.

Participants were strapped to and controlled an inverted pendulum system and the only instruction given was to avoid falls, i.e. maintain the pendulum within the limits of upright balance. Using fall rate as a measure of performance, participants were tested in one session (PRE), then trained in five successive sessions over several days, and then tested again post training (POST).

We devised metrics to quantify the extent to which the FAC strategy and also to which the SC strategy is followed. The FAC metric was the change in correlation of force with position between POST and PRE test sessions. The SC metric was the change in sway between PRE and POST test sessions.

To investigate hypothesis (i) we use regression to test whether the FAC metric is associated with change in performance (falls) and with change in a range of descriptive measures including acceleration, muscle effort and co-contraction.

To investigate hypothesis (ii) we use regression to test whether the SC metric is associated with change in performance (falls) and with change in a range of descriptive measures.

In summary, the aim of this study is to test two hypotheses: (i) FAC is associated with faster learning and fewer falls and (ii) SC is associated with no learning and no reduction in falls.

## Methods

### Ethical approval

The experiments reported in this study were approved by the Academic Ethics Committee of the Faculty of Science and Engineering, Manchester Metropolitan University (EthOS Ref 0567) and conform to the Declaration of Helsinki. Participants gave written, informed consent to the experiment which was performed in the Research Centre for Musculoskeletal Science & Sports Medicine at Manchester Metropolitan University. Participants in Supplementary videos gave consent for publication in online open-access publication.

### Apparatus and balance task

The experiment consisted in a postural balancing task. Participants stood with their feet on a stable footplate and were strapped rigidly to a one degree of freedom actuated device, named Whole Body Mover (WBM). The WBM (Fig. 1) is composed of a vertical board rotating around a joint collinear with the ankles, connected to a direct drive linear actuator (XTA3810S, Servotube Actuator, Copley Motion, UK) at approximately 1 m above the axis of rotation. An incremental position encoder is located in the linear actuator. The starting position of the WBM was set to 2° forward respect to the vertical line, to approximate physiological standing^5^ using an absolute position potentiometer mounted on the rotational axis. The task was implemented using Simulink, compiled using Real-Time Workshop and executed on a PC using Real-Time Windows Target within MATLAB (all from MathWorks, Natick, MA, USA) with a control loop frequency of 1 kHz. Following each recording, all signals were saved at 100 Hz.

A control signal applied as torque to the virtual unstable system was generated by a myoelectric interface connected to the participant ankle muscles. The interface was implemented by a multichannel surface electromyograph (sEMG) (Trigno, Delsys) with a sample frequency of 2 kHz and Ag/AgCl electrodes were used to measure the electrical activity of leg muscles Tibialis Anterior (TA) and calf muscles (intersection of Gastrocnemius Medialis and Soleus (G) of both right and left leg. Electrodes placement was accomplished according to SENIAMs (Surface ElectroMyoGraphy for the Non-Invasive Assessment of Muscles) recommendations^18^. Once the electrodes were in place, the electrical activity in all muscles was recorded while muscles were at rest in order to remove noise due to spontaneous electrical activity, not corresponding to muscle work. Those dead-zone values were measured at the beginning of each experimental session. Throughout the task, sEMG signals were processed in real-time through a low-pass filter (cut-off frequency: 340 Hz) and then rectified. The specific control signal was generated by the sum of the muscular contributions of the two legs evaluated as the sEMG envelops signals difference between the two antagonist muscles (TA and G). The actuated position of the WBM was controlled to follow the output of the real time simulated unstable system using a proportional–integral–derivative (PID) controller. In order to study the process of postural balance, the actuated system had an unstable (inverted pendulum) time constant of a typical human body represented by the transfer function in Eq. 2 ^19^.

2 $$P=\frac{6.977}{({s}^{2}+0.03721\,s-1.231)}$$

Moreover, in the case of human balance, Loram *et al*.^3^ showed that the damping is negligible. Following this evidence, we implemented the system transfer function that is controlled by the human to have negligible damping. The WBM itself, which presents the system output to the participant as the position of the board, was controlled by position control which negated very effectively the mechanical structure of the WBM. The irrelevance of the mechanical structure was evaluated using the cross-correlation between simulated output and measured position of the WBM. Using cross correlation to estimate the delay, during these tasks, the delay between simulated output and measured position of the WBM was 4 ± 3 ms (mean ± S.D.) from all 448 trials, which can be considered negligible respect to the physiological processes involved. If the WBM exceeded a range of motion of ±10° the WBM was deactivated and returned gently to the initial position of 2°, and the task continued automatically after a delay of 5 s. This reset phase was termed “falling over”.

#### Participants and Protocol

Fifteen healthy participants (6 F + 9 M, 33 ± 8 years) took part in the experiment (Table 1). Participants were first prepared for sEMG recording and baseline thresholds were recorded as above. Participants were then strapped to the WBM and given a short familiarisation with the task of approximately 5 mins which was sufficient to feel comfortable with the task. For the balance task, participants were instructed to not ‘fall over’, which was explained as meaning to keep the WBM within the range of motion (±10°). We allowed the maximum range of movement possible: the limit corresponds with the mechanical range of the system. The range was limited by the stroke of linear actuator which moves the board. However the range is compatible with that which applies in quiet symmetrical standing. Forwards, the foot length from the ankle is approximately 0.2 m. For a centre of mass at 1 m height this gives a forward angle stability limit of 0.2/1 radians = 11.45 degrees. Backwards, the heel length from the ankle is approximately 0.08 m. This gives a backward stability limit of 0.08/1 radians = 4.6 degree. Therefore, the range (±10 degrees) corresponds approximately to the natural limits of stability.

**Table 1:** Participants details: sex, age, height and weight.

PARTICIPANT	SEX	AGE(y)	WEIGHT(kg)	HEIGHT(cm)
P1	F	31	53	160
P2	F	26	63	171
P3	F	29	59	167
P4	M	54	67	178
P5	M	27	94	183
P6	M	31	92	186
P7	M	32	80	178
P8	M	47	72	173
P9	F	25	58	164
P10	M	39	60	160
P11	M	34	70	169
P12	F	27	54	164
P13	M	36	75	176
P14	F	36	53	160
P15	M	34	94	180

Merely keeping the WBM within range was in fact a trivial task for participants and one which they learned in a matter of minutes. To ensure the task was challenging an input disturbance was applied and discrete changes to the gain of the myoelectric control signal were also applied.

- A multisine disturbance was added to the control signal and hence to the input of the plant dynamics. The multisine disturbance contained 100 frequency components equally spaced in the range 0.1–10 Hz. For each trial the phases were randomised and the crest factor (ratio of maximum deviation to SD) was limited to 3 making the signal unpredictable but periodic^20^.
- The gain of myoelectric control signal was changed periodically during the task (each period lasted 20 s). This perturbation changes the force output applied to the system from the normal muscle activation. To maintain constant force output, during a change in myoelectric gain, a participant would have to adjust muscle activity inversely to the change in myoelectric gain.

**Figure 3:**
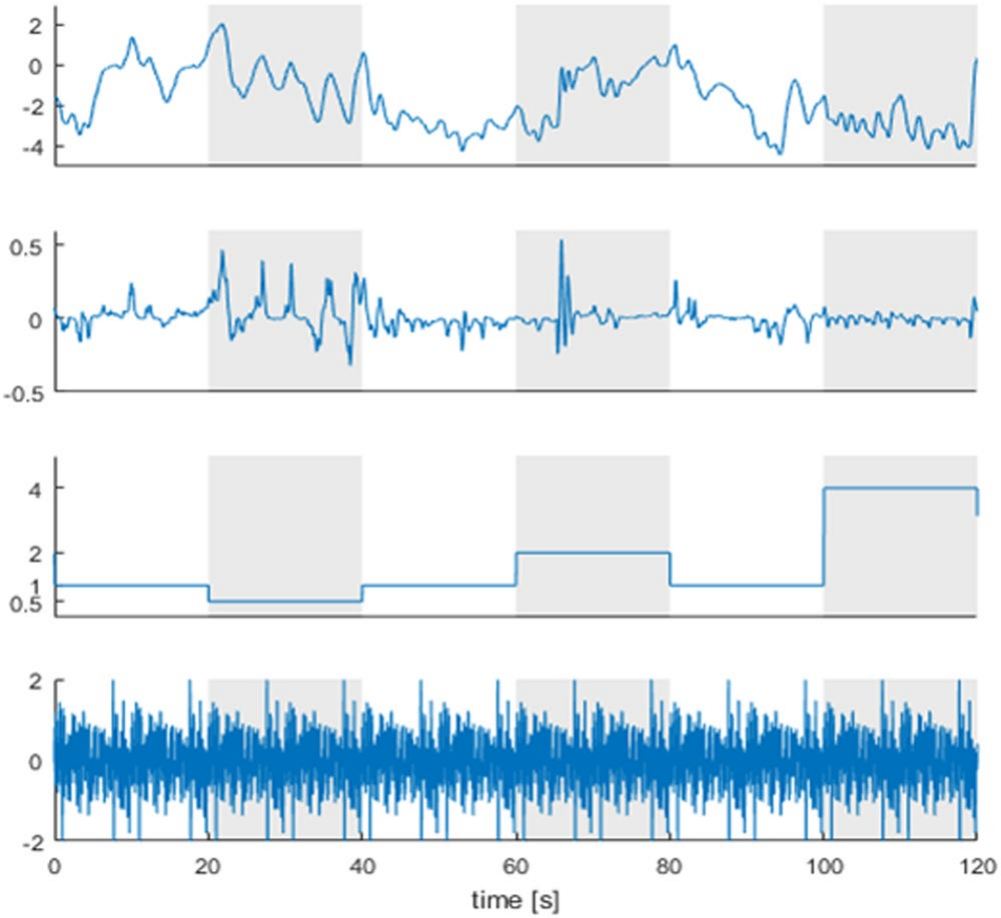
Representative signals recorded during the experiment. Starting from the top, the figure shows the angular position [°], the control signal [mV], the gain applied to the control signal and the multisine disturbance [N]. Vertical bands indicate the trials with constant gain. Without any disturbance or challenge, the task feels trivial and to the participant as if they are “doing nothing”. Adding a disturbance and changes in myoelectric gain forces the participant to engage in the task to prevent themselves from falling over.

Four multisine disturbance amplitudes (1, 2, 3, 4) and four myoelectric gain levels (0.5, 1, 2, 4) were applied (Fig. 3).

Following familiarization, the experiment included 7 sessions. We refer to the first session of the training as PRE and to the last session (session 7) as POST. The 7 sessions were distributed in 4 consecutive days (1st day: PRE and 1 session; 2nd day: 2 sessions; 3rd day: 2 sessions; 4th day: POST). Each session consisted of 64 trials of 20 s duration (around 30 minutes per session), including all disturbance amplitude and myoelectric gain levels randomized.

### Data analysis

The angular position, extracted from the encoder was smoothed using a sixth order Savitzky – Golay filter with a cut-off frequency of 10 Hz, which was also used to estimate the subsequent time derivatives. The control signal and the filtered sEMG signals were normalized by the maximum value of the signal, computed considering trials of each participant during the whole experiment.

To address our hypothesis we designed the following measures:

- Success Time (*ST*) [%]: percentage of the time while successfully balancing (i.e. without falling) respect to the entire duration of the trial. This parameter represents the performance outcome of each participant during each training session.
  The following measures describe the manner of performance:
- Linearity index (*L*): Pearson correlation coefficient computed between the control signal and the angular position. It is computed as a measure of accuracy in mapping force to position over a range of positions.
- Sway (*sway*) [deg]: angular range of variation explored in the antero-posterior direction. It was computed as two times the standard deviation of the angular position.
- Co-contraction index (*CC*) [%]: percentage of the time while co-activating ankle antagonist muscles respect to the duration of the trial when participants succeed in stabilization. We considered a muscle active when overcoming a threshold (30% of the maximum normalized sEMG signal). This parameter was evaluated separately for right and left leg and the mean value of the two sides was considered. The computed indicator allows assessing the level of co-activation in terms of duration, associated with stiffness control.
- Effort index (*E*) [mV]: sum of the root mean square values related to the sEMG signals of each muscle. This measure was computed to quantify the muscular effort exerted.
- Mean Acceleration (*a*) [deg/s2]: Mean angular acceleration. It captures how much the control signal, on average, diverges from its linear dependence on the angle.

For each measure, we averaged the values of all 64 trials to represent the behaviour for each session.

To characterize learning, we computed the change (Δ) in each measure between the first (PRE) session and the last (POST) session.

3 $$\Delta X={X}_{POST}-{X}_{PRE}$$

As shown in Eq. 3, change in measure (ΔX) was calculated as the difference between POST mean value and PRE mean value for each participant. Hence, a positive change means an increase of that measure across training. Conversely a negative change means a decrease.

To quantify the extent to which the FAC strategy is followed we calculate a FAC metric. The FAC metric is calculated as the change in correlation of force with position between POST and PRE test sessions (Δ*L*).

To quantify the extent to which the SC strategy is followed we calculate a SC metric. The SC metric was change in sway between PRE and POST test sessions (Δ*sway*).

**Figure 4:**
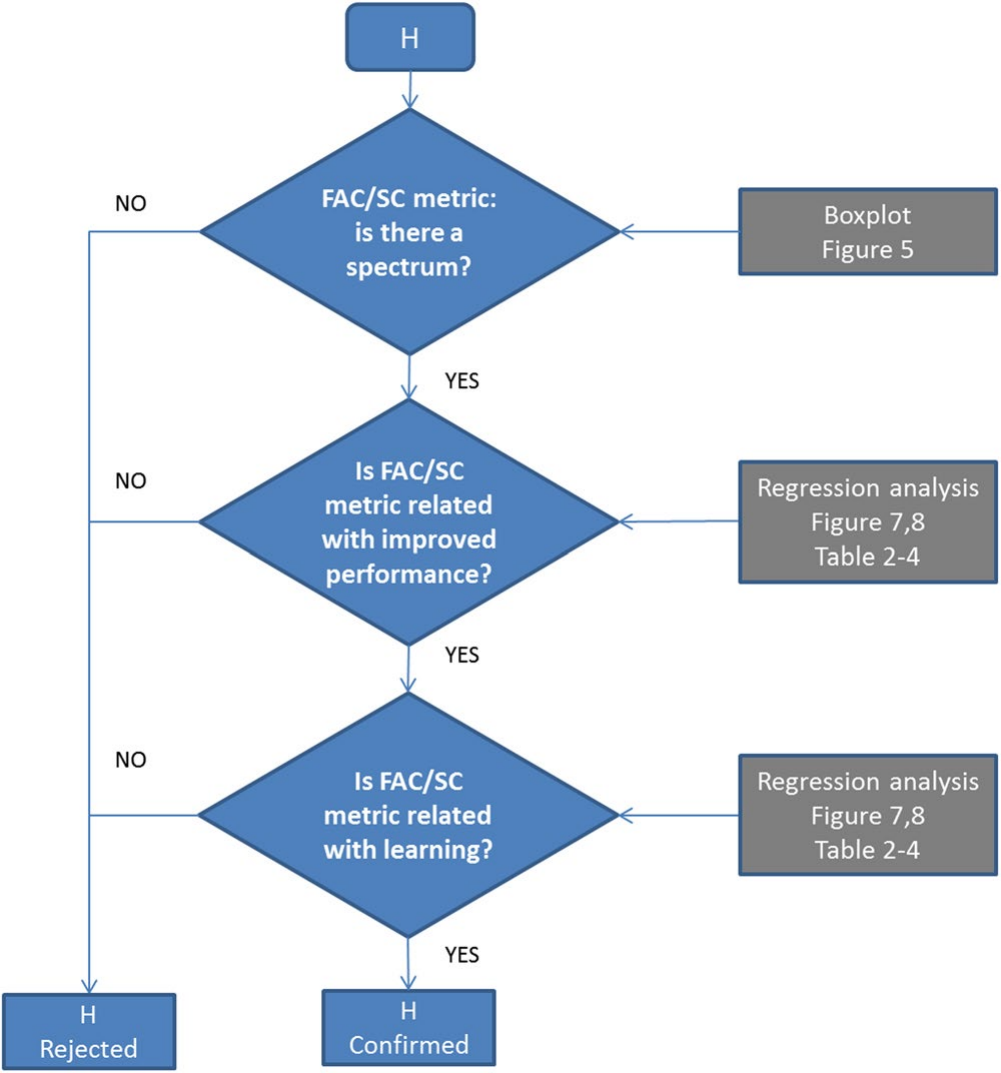
Flowchart to test the two hypotheses (H). Using boxplots we observed the distribution of FAC/SC metrics (Fig. 5). Using regression analysis, we tested for significant correlations between FAC/SC metric number of falls (performance in the instructed task) (Figs. 7, 8 and Tables 2–4). Using regression analysis, we test for significant correlations between FAC/SC metric and manner of performance (learning as indicated by the other measures), (Figs. 7, 8 and Tables 2–4).

Following the flowchart in Fig. 4, we tested our two hypothesis through the following steps: (i) we determined whether there was a spectrum of strategies (positive to negative) observing the distribution of FAC (Δ*L*) and SC (Δ*sway*) metrics, (ii) we used regression analysis to assess learning (change in measures) in relationship with FAC/SC metric and (iii) we used regression analysis to characterize performance (falls) related to FAC/SC metrics. The statistical significance threshold for regression analysis was set at P = 0.05.

## Results

### Distribution of strategies

**Figure 5:**
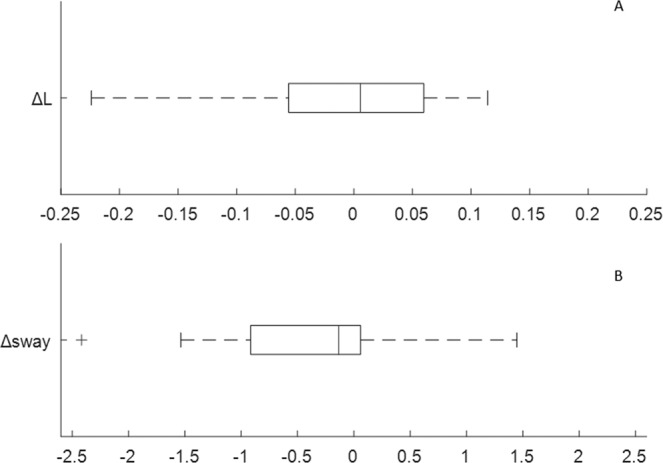
Distribution of strategy metrics of the participants: (**A**) FAC metric (**B**) SC metric. In each box plot the central mark indicates the median, and the left and right edges of the box indicate the 25th and 75th percentiles, respectively. The whiskers extend to the most extreme data points not considered outliers. The outliers are plotted individually using the ‘+’ symbol. For each metric, there was a range of participants including those who changed positively and those who changed negatively.

**Figure 6:**
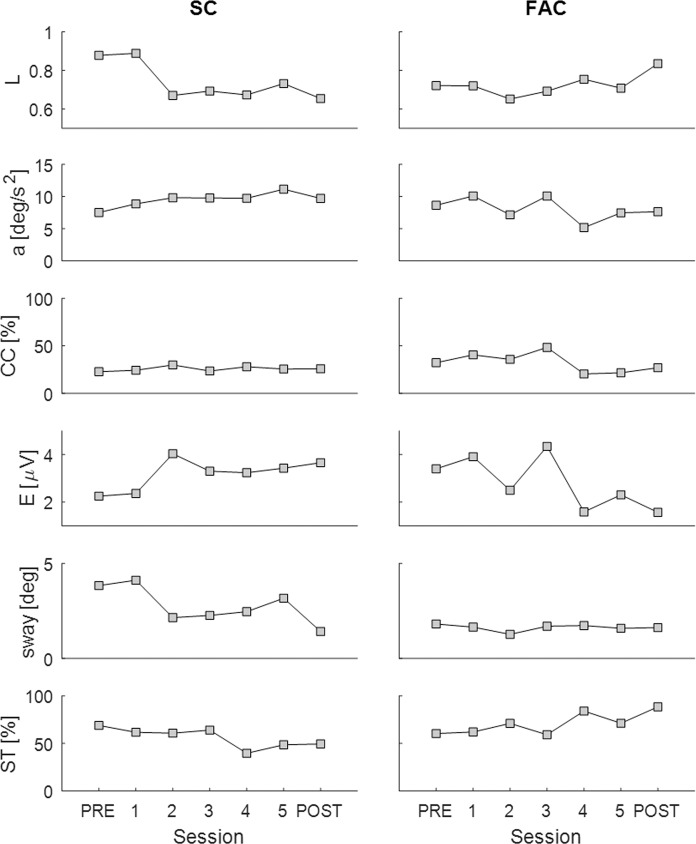
Two representative examples of the trend of the measures of performance and manner of performance throughout practice. In each plot, the x axis represents the sessions and the y axis represents one of the measures. Starting from the top, the measures illustrated are: correlation (L), acceleration (a), co-contraction (CC), effort (E), sway and success time (ST). Left panels shows the trend for one participant adopting a control strategy closer to Stiffness Control, whereas right panels show the behaviour of one participant adopting a control strategy closer to Force Accuracy Control.

This sample of participants shows a spectrum of positive and negative values of both the force accuracy strategy (FAC metric, Δ*L*) and the stiffness control strategy (SC metric, Δ*sway*) (Fig. 5). Two representative examples are shown in Fig. 6. For completeness, the PRE, practice and POST results are shown for all participants in Supplementary Material (Supplementary Figs. 1–15). The inter quartile ranges (IQR) cross zero (Δ*L*: IQR = 0.1153; Δ*sway*: IQR = 0.9752) and the median change is close to zero (Δ*L* = 0.006; Δ*sway* = −0.135 deg) for the FAC and SC strategy respectively (Fig. 5). These distributions ensure we can examine the relationship between increasing or decreasing FAC metric and SC metric on performance (falls).

### Effect of force accuracy control (FAC) and stiffness control (SC) strategy on performance (falls)

The hypothesis to be verified is whether the strategy is associated with a change in performance, i.e. the instructed outcome which is to prevent falls.

**Figure 7:**
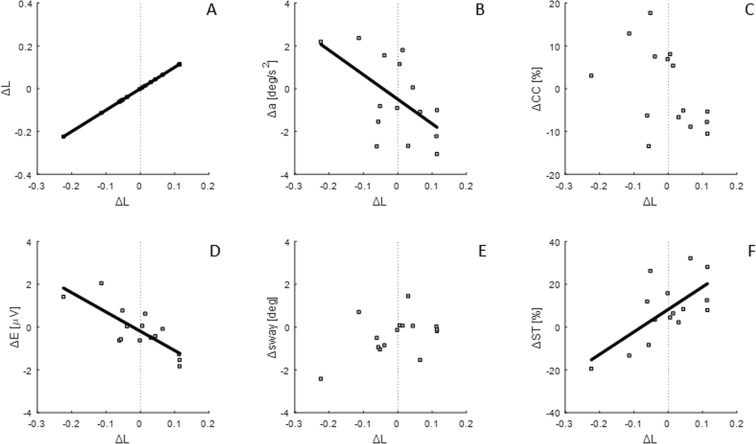
Linear regressions of FAC metric with: (**A**) FAC metric, (**B**) change in acceleration, (**C**) change in co-contraction, (**D**) change in effort, (**E**) SC metric (**F**) change in success time percentage. Each subplot represents data points (grey squares) and the regression line, when correlation is significant. The vertical dotted line corresponds to change in metric equal to zero.

For the participants of this study, the force accuracy strategy (FAC) was associated with a reduction in falls. Specifically, as a result of training, between PRE and POST test sessions, a change in percentage of success time (Δ*ST*) is correlated positively with a change in linearity index (Δ*L*), (R = 0.67, P = 0.006, Table 2, Fig. 7E), reflecting that an improvement in force accuracy is associated with reduced falls.

**Table 2:** Regression analysis of FAC and SC metric with: FAC metric, change in acceleration, change in co-contraction, change in effort, SC metric and change in success time percentage.

	Δ*L*	Δ*sway*
Δ*L*	R = 1; P = 0	R = 0.445; P = 0.097
Δ*a*	R = −0.567; P = 0.027	R = −0.214; P = 0.444
Δ*CC*	R = −0.472; P = 0.076	R = −0.021; P = 0.941
Δ*E*	R = −0.7903; P = 0.0005	R = 0.208; P = 0.45
Δ*sway*	R = 0.445; P = 0.097	R = 1; P = 0
Δ*ST*	R = 0.67; P = 0.006	R = 0.008; P = 0.978

All R and P values are reported.

**Figure 8:**
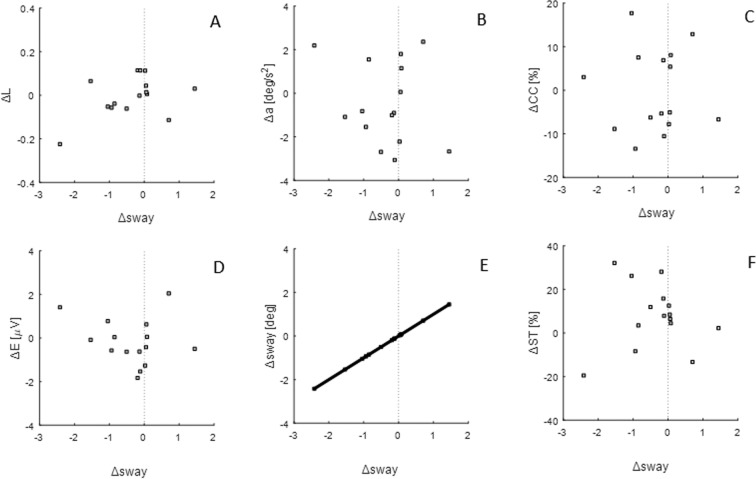
Linear regressions of SC metric with: (**A**) FAC metric, (**B**) change in acceleration, (**C**) change in co-contraction, (**D**) change in effort, (**E**) SC metric (**F**) change in success time percentage. Each subplot represents data points (grey squares) and the regression line, when correlation is significant. The vertical dotted line corresponds to change in metric equal to zero.

Moreover, the stiffness control strategy (SC) was not associated with any change in falls. There is no significant correlation between change in sway (Δ*sway)* and change in percentage of success time (Δ*ST)*, (R = 0.008, P = 0.978, Table 2, Fig. 8E), i.e. increase in sway is not associated with a reduction in falls.

Finally, force accuracy and stiffness control metrics were not correlated with the performance measure (*ST*) calculated in the PRE test session (Δ*L*: R = −0.032, P = 0.909; Δ*sway*: R = 0.282, P = 0.308) (Table 3). After training, force accuracy metric was positively correlated with the performance measure (*ST*) calculated in the POST test session (R = 0.612, P = 0.015), whereas stiffness control metric did not correlate with POST *ST* (R = 0.219, P = 0.432) (Table 4).

**Table 3:** Regression analysis of FAC and SC metric: FAC metric, acceleration, co-contraction, effort, SC metric and success time percentage relative to the PRE session.

	Δ*L*	Δ*sway*
PRE *L*	R = −0.614; P = 0.015	R = −0.299; P = 0.279
PRE *a*	R = 0.14; P = 0.618	R = 0.091; P = 0.746
PRE *CC*	R = 0.375; P = 0.169	R = 0.112; P = 0.692
PRE *E*	R = 0.424; P = 0.115	R = −0.118; P = 0.674
PRE *sway*	R = −0.386; P = 0.156	R = −0.705; P = 0.003
PRE *ST*	R = −0.032; P = 0.909	R = 0.282; P = 0.308

All R and P values are reported.

**Table 4:** Regression analysis of FAC and SC metric: FAC metric, acceleration, co-contraction, effort, SC metric and success time percentage relative to the POST session.

	Δ*L*	Δ*sway*
POST *L*	R = 0.582; P = 0.023	R = 0.232; P = 0.406
POST *a*	R = −0.447; P = 0.094	R = −0.14; P = 0.618
POST *CC*	R = −0.161; P = 0.567	R = 0.068; P = 0.809
POST *E*	R = −0.655; P = 0.008	R = −0.31; P = 0.261
POST *sway*	R = 0.177; P = 0.527	R = 0.584; P = 0.022
POST *ST*	R = 0.612; P = 0.015	R = 0.219; P = 0.432

All R and P values are reported.

Hence results are consistent with our first hypothesis. Positive change in FAC metric is associated with improved performance. The second hypothesis is contradicted. Neither positive change in SC metric is associated with improved performance, nor is negative change in SC metric associated with poor performance.

### Effect of force accuracy control (FAC) and stiffness control (SC) on the manner of performance (effort, acceleration)

While instructed performance is measured by falls, the further aspect of the hypothesis to be verified is whether the strategy is associated with a change in manner of performance. The force accuracy strategy (FAC) was associated with a reduction in effort and in acceleration. As a result of training sessions, between PRE and POST test sessions, change in effort (Δ*E)* and change in acceleration (Δ*a*) are correlated negatively with a change in the linearity index (Δ*L*), (Δ*E*: R = −0.7903, P = 0.0005*;* Δ*a*: R = −0.567; P = 0.027) (Table 2, Fig. 7E), i.e. an improvement in force accuracy is associated with reduced effort and reduced acceleration.

Moreover, the stiffness control strategy (SC) was not associated with any change in the manner of performance. There is no significant correlation between change in sway (Δ*sway*) and change in effort (Δ*E*: R = 0.208, P = 0.45) or change in acceleration (Δ*a*: R = −0.214, P = 0.444) (Table 2, Fig. 8E), i.e. a change in sway is not associated with a change in effort and acceleration.

Finally, force accuracy and stiffness control metrics were not correlated with any of the descriptive measures nor the performance measure calculated in the PRE test session (Table 3). After training, force accuracy metric was positively correlated with POST *L* (R = 0.582, P = 0.023) and negatively correlated with POST *E* (R = −0.655; P = 0.008) calculated in the POST test session, whereas stiffness control metric did not correlate with any measure (Table 4).

Hence our first hypothesis is confirmed: Force accuracy control strategy is associated with learning to improve performance and reduce force error, minimizing acceleration and effort. Our second hypothesis is rejected: Stiffness control strategy is not associated with change in performance or change in manner of performance.

In conclusion, our first hypothesis that Stiffness Control is associated with learning and better performance was rejected.

Our second hypothesis that Force Accuracy Control is associated with faster learning and better performance was confirmed.

## Discussion

This study used a challenging postural task, to investigate the relationship between the control strategy adopted to maintain balance and the level of learning and robustness to falls.

Our hypotheses were (i) that minimising force error (FAC) maximizes accurate mapping of the force vs position relationship and it is associated with faster learning and fewer falls; and (ii) that minimising sway (SC) reduces exploration of system properties such as the force vs position relationship and is associated with poor learning and limited reduction in falls. We devised measures descriptive of the strategies and tested those for correlation with measure of performance and manner of performance. The results confirm the predicted associations, however, interpreting the relevance of the preceding theory requires some discussion.

Results confirm that as a group, participants maintained balance using a spectrum of control strategies (Fig. 5). We show that prioritizing regulation of force (FAC) was correlated with better learning and better performance (falls reduction) (Fig. 7), whereas regulation of position (SC) was not (Fig. 8). Specifically, increased correlation between force and position (representing an improvement in force accuracy, Δ*L* > 0), was associated with improvement in performance (*ST*) (Fig. 7E), and decreased correlation between force and position, representing a deterioration of force accuracy (Δ*L* < 0), was associated with poorer performance (Fig. 7E). By contrast, minimising sway (SC) was not associated with change in any measures except sway and was not associated with reduction in falls (Fig. 8). Sway was unrelated to performance (falls) and manner of performance (effort, co-contraction, acceleration) (Fig. 8, Tables 2, 3).

The SC and FAC metrics were uncorrelated, removing possible evidence of association between exploration of position and ability to maintain an appropriate torque for the position of the inverted pendulum. A correlation between reduction in sway and decreased performance, or manner of performance, would have supported the hypothesis that sway allows acquisition of information important for learning^15^. However, the absence of such a correlation does not refute the exploration hypothesis. We have also to consider that the FAC metric denotes ability to correlate force with position over a range of positions of an unstable system. FAC is thus equivalent to combining low force error (low acceleration) with wide range of positions. Hence the exploration hypothesis remains neither supported nor refuted.

Since force minimization (FAC) is linearly related to fall rate, whereas position minimization (SC) is not, the variable FAC is identified as more important than position for performance (falls) and manner of performance (effort, acceleration). Increased FAC represents a combination of reduced acceleration and increased sway, including also reduced acceleration and constant sway, or unchanged acceleration and increased sway. The importance of FAC is in line with a computational study^21^ showing that once the position, velocity and acceleration (which is proportional to force error) are estimated in an optimal way, the proportional– derivative–acceleration (PDA) controller provides better stability properties than the corresponding proportional– derivative (PD) controller.

However, given physiological delays, a PDA controller has to predict the actual state based on the delayed position, velocity and acceleration. Prediction accuracy is relevant to performance. Accuracy in the implicit perception of the torque vs angle relationship of the inverted pendulum is crucial to the relationship between motor commands (force) and motion, enabling the central nervous system to adapt the dynamics of the body to the environment. The observed correlation of increasing force accuracy control (FAC) with decreasing effort (Fig. 7D), combined with no correlations with sway minimization (SC) (Fig. 8, Table 3), supports the hypothesis that learning is related to force accuracy. Participants who improved force accuracy improved their performance and reduced their effort. This relationship is consistent with previous works showing how control becomes more economical as the dynamics of the task are learned^22–25^.

Similarly, the change in acceleration correlated negatively only with FAC metric; i.e. force accuracy and learning are associated with minimization of acceleration more than minimisation of sway (Figs. 7B, 8B). Experimental and modelling studies on quiet standing suggest that the goal of the central nervous system is not to keep the centre of mass at a constant position, but rather to minimize its acceleration^2,26^.

Concerning muscles co-contraction, we expected to find *CC* positively correlated with the SC metric, supposing that minimization of position variance (stiffness control) is achieved by co-contraction of the antagonist muscles similarly to upper limb. However, co-contraction did not correlate with any of the strategy metrics (Tables 2–4). The lack of association (*CC* vs SC) is reasonable because in natural standing, ankle stiffness is not enhanced by co-contraction due to the low series stiffness of the tendons crossing the ankle joint^3,4^. Likewise in this experiment co-contraction generated no passive change to the computer controlled dynamics of the inverted pendulum.

It remains unclear why participants adopt one strategy rather than another. During the PRE session, all participants started from similar conditions, since neither FAC nor SC showed any correlation with any measures of performance (Table 3). Furthermore, we found no correlation between any anthropometric features (height, mass) or age and the strategy adopted.

The aim of this study was to identify the quantities regulated in the control. In the optimal feedback control framework the choice of the control strategy is represented within the cost function as prioritizing regulation of position or regulation of effort.

In practice the use of forward models produces results which are very similar to pure state feedback^27^. An intermittent type controller produces results which are also very similar to control using a forward model or using state feedback^27^. In fact we can say that intermittent control masquerades as continuous control when the open loop predictions are well matched to an underlying, continuous closed loop system^28,29^. This experiment is not designed to discriminate intermittent from continuous control. Discrimination of intermittent from continuous control requires identification of a refractory period or open loop interval^30,31^. Thus, in this paper we avoid discussion of the control law and focus on the main result, which is agnostic as to whether the control law uses a forward model, pure state feedback or intermittent control.

Certainly the proposed task has similarities and differences with upright standing. Similar to normal standing, the participant is in an upright position standing upon their feet, using their calf and tibialis anterior muscles to control the small forward and backward movement of their own body that are similar in size to normal standing. Thus the task feels quite natural. Similar to normal standing, the task requires the participant to keep the horizontal position of the center of mass within a limited range, close to the normal range of the base of support. We apply a continuously varying force perturbation, thus this task requires the participants to balance an unseen external force, while keeping the centre of mass position within the limited range. Thus this task tests the same processes as required for natural standing subject to an external force perturbation, namely control of a single output variable (position of centre of mass), while rejecting an external force disturbance.

Different to normal standing, the task has only one degree of control (the ankle joint) as opposed to the multiple degrees of control (ankle, knee, hip, etc.) during natural standing. Thus, with fewer muscles and joints available this task is slightly harder than natural standing since the participant has fewer options for dealing with a force perturbation.

Since the process tested (control of a single output variable), while perceiving and balancing an external force, the same as perturbed natural standing, we argue our results are relevant to natural standing and the task does not mislead the inference.

Concerning the goal of the task, the instruction was “to avoid falls”. Since the only instruction given to all participants was “to avoid falls”, the instruction did not prejudice the question which concern the strategy used to avoid falls. Thus, we suggest the instruction or goal does not bias the result or mislead the inference.

In conclusion, this study showed that the adoption of a force accuracy control strategy led the participants to better performance, better energetic efficiency and better learning, independently on their control strategy on position. From an optimal motor control perspective, the strategy is represented by the choice of the cost function to minimize^32^: our data suggest that prioritising regulation of force leads to better learning.

Since our FAC metric predicts reduced falls and more economical balance in a natural context, we propose this measure as a potential clinical indicator and also as a potential measure for feedback to guide training to improve balance. A number of studies conducted on athletes and elderly people suggest that decreased muscles activation and lower joint stiffness levels prevent muscle fatigue, falls and injuries^33,34^. Our evidence is limited to 15 participants, but the results support further investigation of use of this metric to train people to change their strategy and avoid potential falls or injuries in everyday life situations. This metric, and possibly also this experimental regime, might prove beneficial for several populations - ranging from young athletes to elderly people with impaired balance, but also Parkinson’s disease patients or stroke survivors.

## Supplementary information

Supplementary Information.

Supplementary Video 1.

Supplementary Video 2.

## Data Availability

The authors confirm that all data supporting the findings of this study are available within this manuscript and its Supplementary Materials. However, the raw data are also available upon request.
