# Supplementary material for: Force accuracy rather than high stiffness is associated with faster learning and reduced falls in human balance
Source: Sci Rep. 2020 Mar 18;10:4953. doi: 10.1038/s41598-020-61896-1 (PMC7080839; doi:10.1038/s41598-020-61896-1)
Supplement: Supplementary file 1 — Supplementary Information. [file 41598_2020_61896_MOESM1_ESM.docx]

**Force accuracy rather than high stiffness is associated with faster learning and reduced falls in human balance**

Amel Cherif^1,2*^, Ian Loram^3^, Jacopo Zenzeri^1^

^1^Department of Robotics, Brain and Cognitive Sciences, Istituto Italiano di Tecnologia, Via Enrico Melen, 83, 16152, Genoa, Italy

^2^Department of Informatics, Bioengineering, Robotics, and System Engineering, University of Genoa, Via All'Opera Pia, 13, 16145, Genoa, Italy

^3^Cognitive Motor Function Research Group, Research Centre for Musculoskeletal Science & Sports Medicine, Dept of Life Sciences, Faculty of Science and Engineering, Manchester Metropolitan University, Manchester, M1 5GD, UK

**^*^Corresponding author:**

Via Enrico Melen, 83, 16152 Genova GE

amel.cherif@iit.it

[ORCID: 0000-0002-1531-6943](https://orcid.org/0000-0002-1531-6943)

**SUPPLEMENTARY INFORMATION**

This paper has supplemental material.

**Supp Video 1 Illustration of motion and actuation of the apparatus.** The apparatus (WBM) is actuated myoelectrically. In this video, sEMG signals from two arm muscles are acquired and used to generate the control signal. This signal drives an unstable, second order system (virtual inverted pendulum). The real WBM is actuated by a direct drive linear servotube to rotate around its single axis of rotation. The position of the WBM is controlled to follow the position of the virtual inverted pendulum with a delay of approximately 4 ms. With a longer delay, the position is displayed visually on the screen by the green ball. In this example, for visibility, contraction of deltoid makes the WBM lean forwards; activation of the triceps makes the WBM lean backwards. This video illustrates the process of controlling the WBM using myoelectric signals. Our experimental setup, which mimics postural balance, is shown in Supp Video 2.

**Supp Video 2 Illustration of experimental setup.** The participant who is strapped to the apparatus (WBM) stands upright with their feet on horizontal surface fixed to the ground. The WBM is a single segment board which rotates around a single axis of rotation aligned approximately with the human ankle joints. The WBM is actuated myoelectrically: sEMG signals from Tibialis Anterior and Calf muscles in both legs are acquired and used to generate the control signal representing a combined net ankle torque. The control is myoelectric only and contains no passive component. The WBM provides the participant with haptic feedback, natural visual feedback and natural vestibular feedback of motion of the unstable second order system (virtual inverted pendulum with time constant of an adult human) rotating around the real axis of rotation in line approximately with the participant ankle joints.

**Supp Figure 1-15** Trend of the measures of performance and manner of performance throughout practice for each participant (P1-P15). In each plot, the x axis represents the sessions and the y axis represents one of the measures. Starting from the top, the measures illustrated are: correlation (L), acceleration (a), co-contraction (CC), effort(E), sway and success time (ST).

**
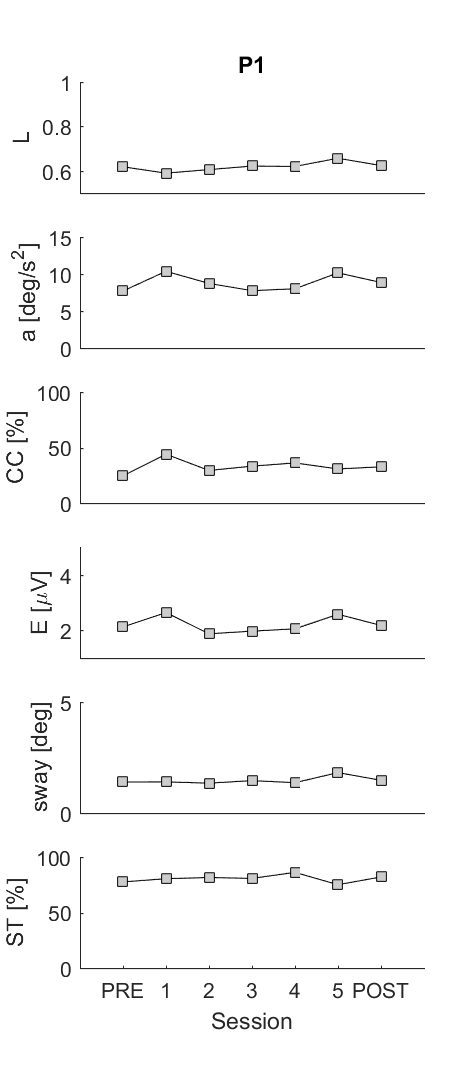

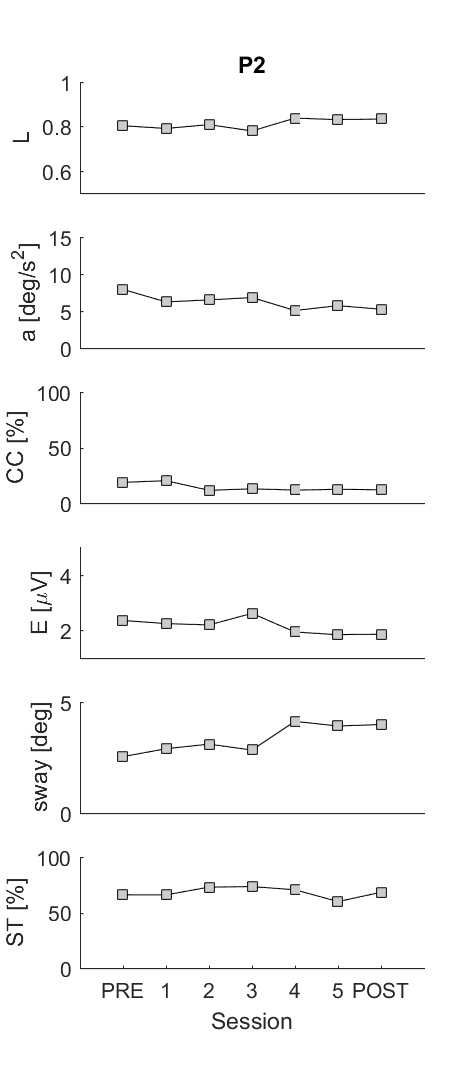

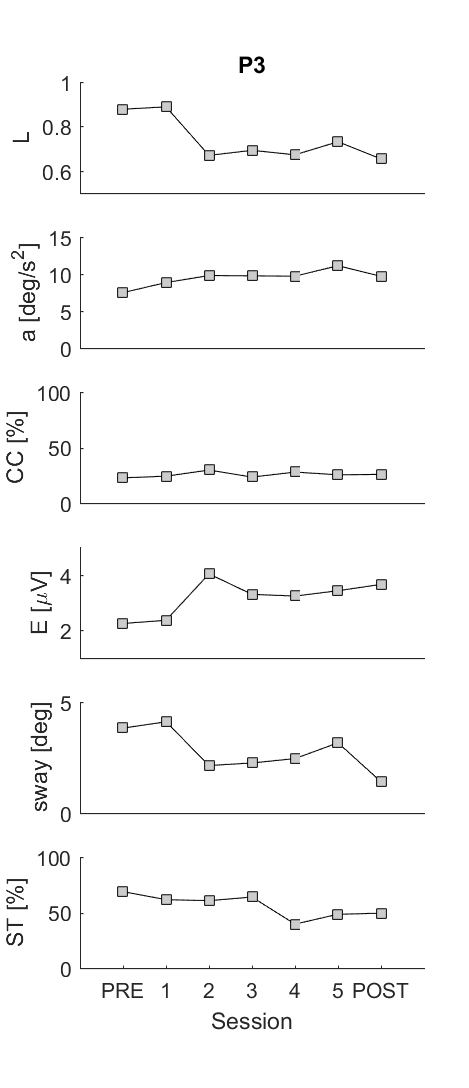

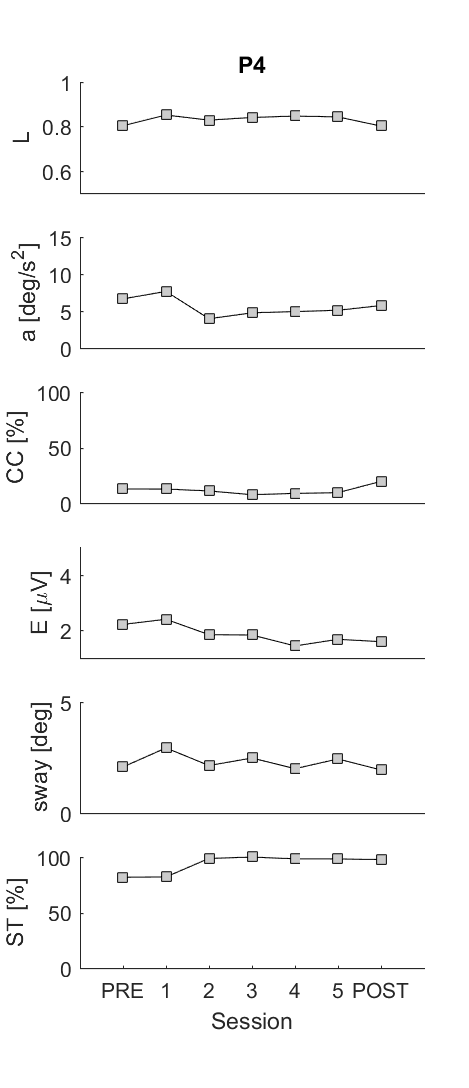

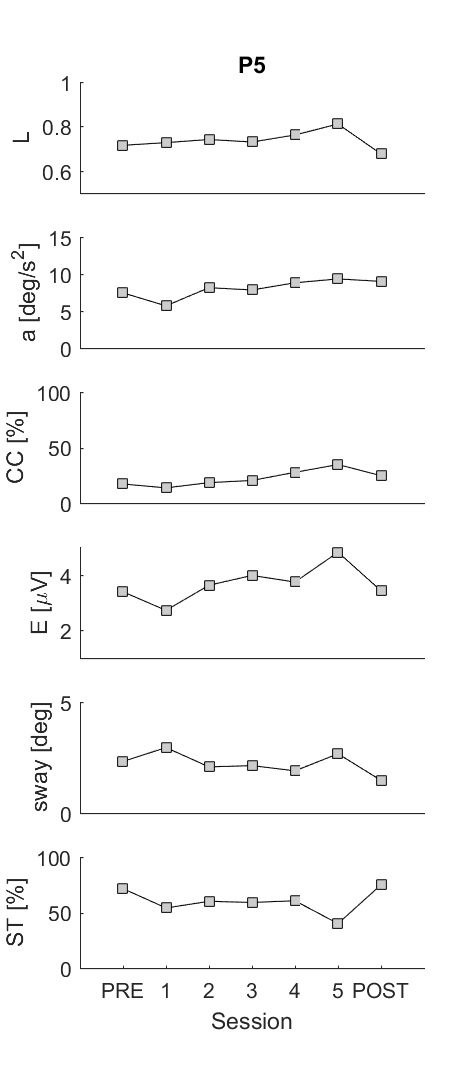

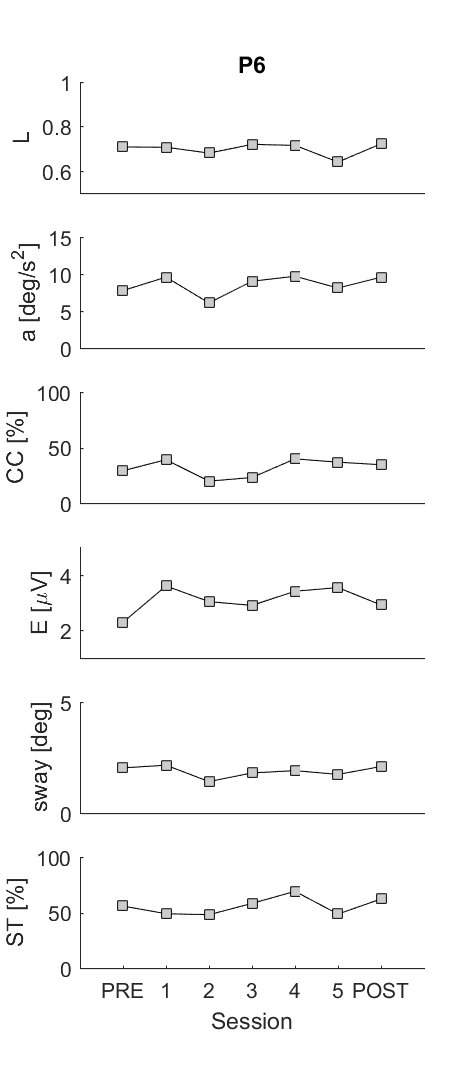

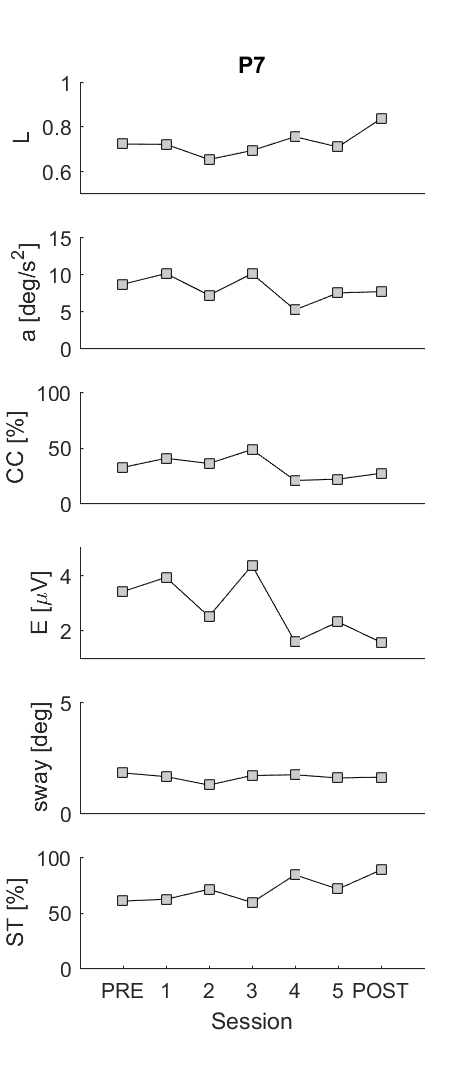

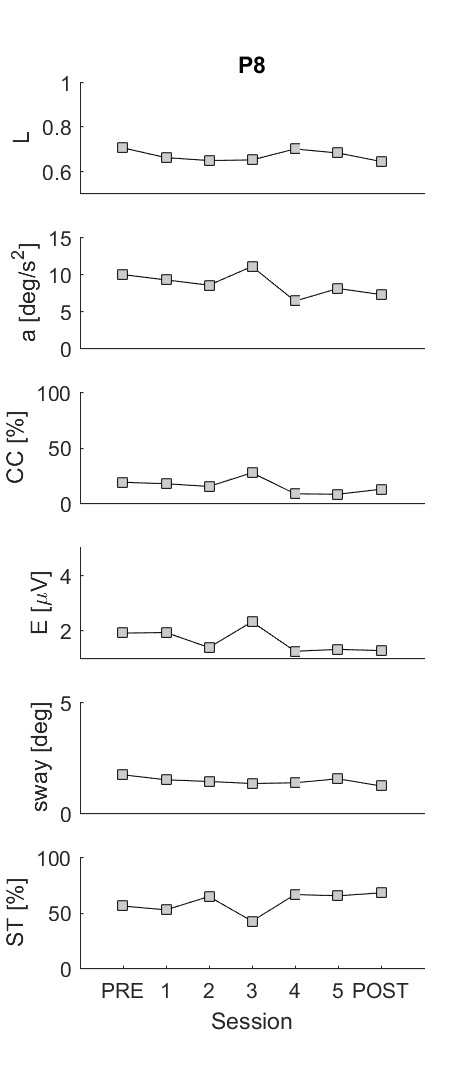

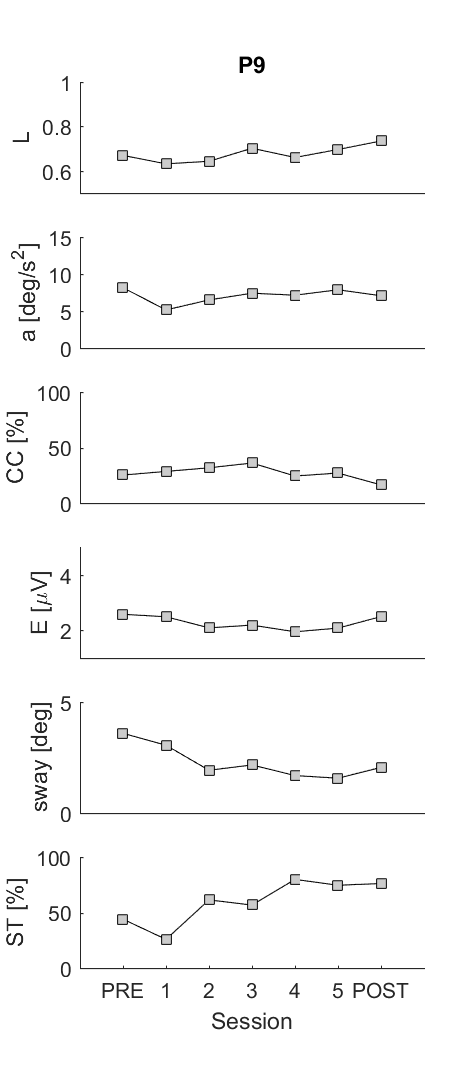

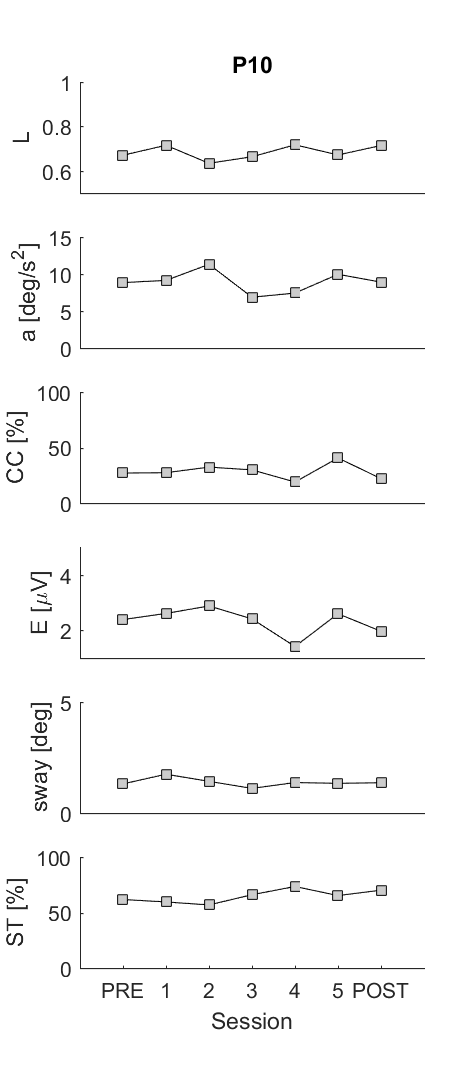

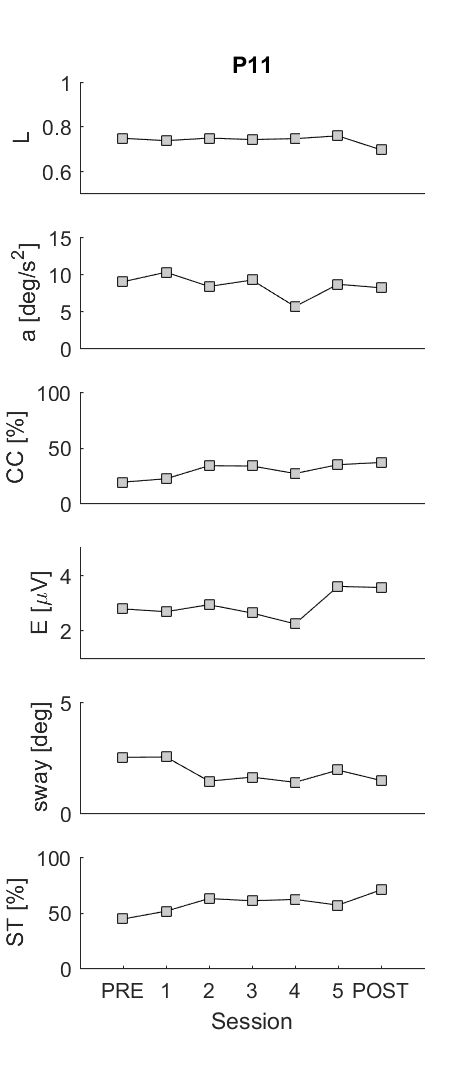

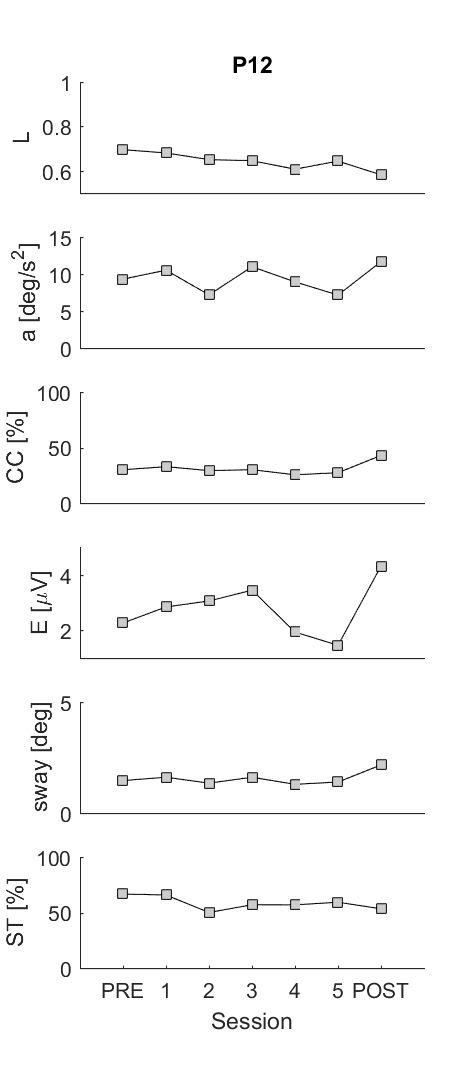

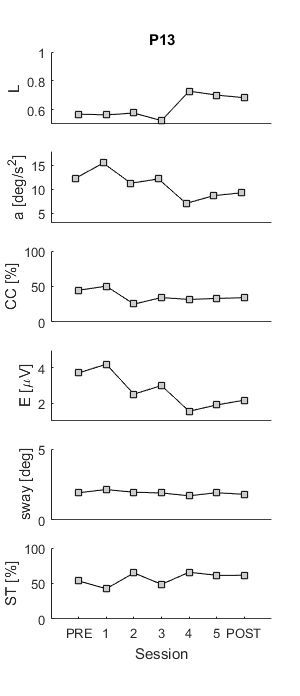

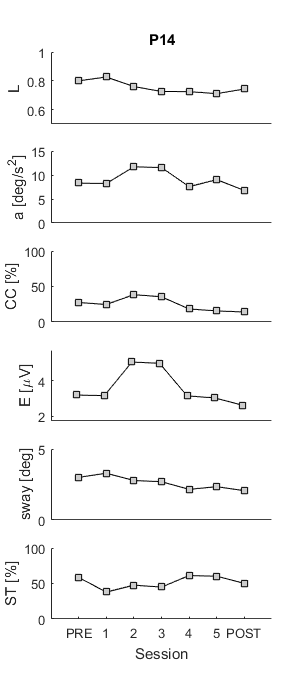

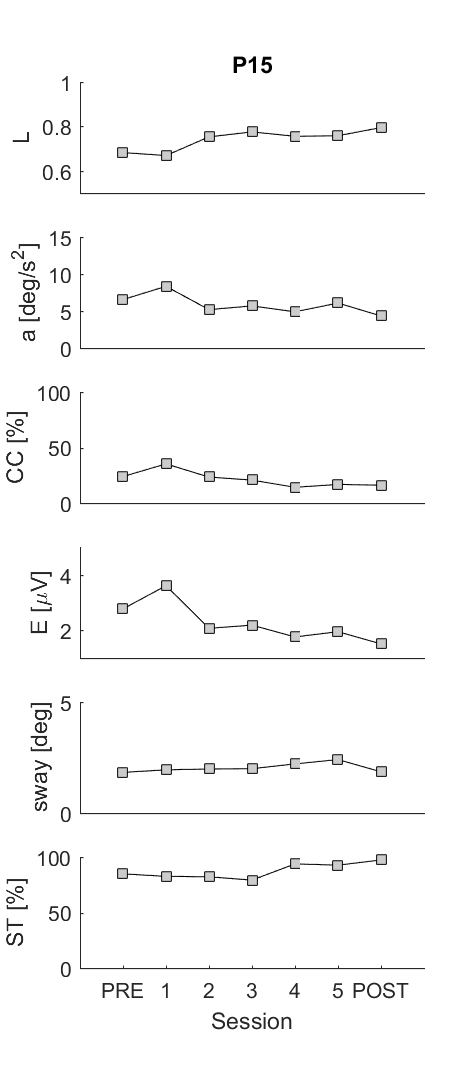
**
